# Effect of Carbon Nanotubes on the Na^+^ Intercalation Capacity of Binder Free Mn_2_V_2_O_7_-CNTs Electrode: A Structural Investigation

**DOI:** 10.3390/ma16052069

**Published:** 2023-03-02

**Authors:** Rahul Parmar, Javad Rezvani, Matteo Amati, Luca Gregoratti, Decio Batista de Freitas Neto, Jose Mauricio Rosolen, Roberto Gunnella

**Affiliations:** 1 Elettra-Sincrotrone Trieste, Strada Statale 14, AREA Science Park, 34149 Trieste, Italy; 2 Physics Division, School of Science and Technology, Università di Camerino, Via Madonna delle Carceri 9, 62032 Camerino, Italy; 3 Departamento de Química, Faculdade de Filosofia, Ciências e Letras de Ribeirão Preto, Universidade de São Paulo, Avenida Bandeirantes 3900, Ribeirão Preto 14040-901, SP, Brazil

**Keywords:** MVO-CNTs composite, CNTs mass loading, X-ray imaging, cathode electrolyte interphase (CEI), binder-free NIBs electrode

## Abstract

Improvements in sodium intercalation in sodium cathodes have been debated in recent years. In the present work, we delineate the significant effect of the carbon nanotubes (CNTs) and their weight percent in the intercalation capacity of the binder-free manganese vanadium oxide (MVO)-CNTs composite electrodes. The performance modification of the electrode is discussed taking into account the cathode electrolyte interphase (CEI) layer under optimal performance. We observe an intermittent distribution of the chemical phases on the CEI, formed on these electrodes after several cycles. The bulk and superficial structure of pristine and Na${}^{+}$ cycled electrodes were identified via micro-Raman scattering and Scanning X-ray Photoelectron Microscopy. We show that the inhomogeneous CEI layer distribution strongly depends on the CNTs weight percentage ratio in an electrode nano-composite. The capacity fading of MVO-CNTs appears to be associated with the dissolution of the Mn${}_{2}$O${}_{3}$ phase, leading to electrode deterioration. This effect is particularly observed in electrodes with low weight percentage of the CNTs in which the tubular topology of the CNTs are distorted due to the MVO decoration. These results can deepen the understanding of the CNTs role on the intercalation mechanism and capacity of the electrode, where there are variations in the mass ratio of CNTs and the active material.

## 1. Introduction

Sodium-ion batteries (NIBs) are currently becoming commercial and their usage in several devices is expected to grow in the near future, thus explaining the number of studies aiming to identify new electrode materials for NIBs [1,2,3]. In this context, the formation of protective layer electrodes such as the cathode electrolyte interphase (CEI) layer resulting from electrolyte decomposition plays a relevant role in the performance of intercalation batteries (e.g., life span and safety) [4,5,6]. Researchers have been investigating the development of new cathode materials for high-performance NIBs; however, electrode–electrolyte interphase chemistry remains less studied [7,8,9,10,11]. The formation of a cathode electrolyte interphase (CEI) layer after certain charge–discharge cycles affecting the NIB’s lifespan and performance has been reported so far in the literature [6,12,13]. The determination of the chemical composition of CEI with high-resolution spectroscopies can be useful to the design of electrodes, electrolytes, and their additives whereby the Coulombic efficiency becomes stable after several discharge/charge cycles. It is well known that solid electrolyte interphase (SEI) on the anode and CEI layer on the cathode sustains different thickness and provokes potential differences [14]. Generally, the CEI layer thickness decomposed from sodium hexafluorophosphate–propylene carbonate (NaPF${}_{6}$-PC)-based electrolyte may vary within the range of several nanometers, i.e., 10–30 nm [15,16]. The chemical species of the CEI layer can be different in bulk (mean probing depth (MPD) ≥ 10 nm) or on surface (MPD ≤ 1 nm) [17,18]. An increase in CEI layer thickness creates an impedance between electrolyte–electrode interphase and thus presents a bottleneck for Na${}^{+}$ ion kinetics. Iqra Moeez et al. reported that the addition of artificial Na${}_{2}$CO${}_{3}$ and NaF layer components with conventional electrolyte provided an improvement in NIBs performance [19]. The discontinuous CEI layer has been observed to endure defects on the electrode material surface due to acid attack and facilitation of transition metal oxide dissolution [20]. To protect the electrode materials from acid attack and a thick layer of electrolyte decomposition, several techniques have been proposed such as encapsulation by an amorphous material (for example, Al${}_{2}$O${}_{3}$), reduction in nanoparticle size, forming composite with carbon nanotubes (CNTs), graphene oxide (GO), conductive polymers, doping of a foreign element and electrolyte-modification engineering [18,21,22].

In this research, we employed the CNTs and manganese vanadium oxide (MVO) also known as manganese pyrovanadate-composite-based electrode to achieve high NIBs performance. The crystal structural formula of MVO is defined as A${}_{2}$B${}_{2}$O${}_{7}$, where A (Mn) and B (V) are divalent and pentavalent, respectively. MVO shows a distorted honeycomb-like atomic structure (monoclinic (c2/m) phase) where the oxygen anions, manganese (Mn${}^{2+}$) and vanadium (V${}^{5+}$) cations are arranged in hexagonal close-packed sites, octahedral and tetrahedral sites in alternating parallel layers [23]. Manganese–vanadium oxide composite shows low electrical conductivity, representing one of the reasons behind the poor electrochemical performance of MVO-based NIBs. Recently, C. Zheng et al. reported an extremely stable discharge specific capacity of 120 mA h g${}^{-1}$ at a high current density (20 A g${}^{-1}$) sustained for 5000 charge–discharge cycles for a single crystal MVO with conductive carbon black and polytetrafluoroethylene-binder-based electrode [24].

Therefore, MVO can be considered as a suitable candidate for long-life NIBs application; however, it still bears low discharge capacity due to structural instability and CEI growth. For this reason, we used CNTs to help improve the electrical conductivity and mechanical stability of the electrode, i.e., MVO-CNTs composite on carbon fiber (CF) where no organic binder and current collector were used in electrode fabrication. This robust MVO-CNTs-CF binder-free electrode has the ability to replace aluminium and/or copper current collector which may reduce overall weight, cost and make NIBs even more flexible for future wearable electronic device application. An appropriate weight percentage (wt.%) or mass loading of CNTs mixed with metal oxide nano/micro-structured material requires optimization for improved NIBs performance and lifespan. Thus, we aim to draw the particular attention of researchers from the NIB’s scientific community to focus on the optimization of mass loading ratio of active, inactive electrode material’s wt.% and additive’s wt.% for optimal battery performance. In this article, we detail the electronic structural properties and chemical phase distribution, i.e., CEI layer using high-resolution X-ray Scanning Photoelectron Microscopic mapping technique on Na${}^{+}$ cycled MVO-CNTs-CF composite material and perform a comparison with pristine MVO-CNTs-CF electrode results. Herein, we establish an imperative relation between wt.% ratio of CNTs, electrochemically deposited MVO material on CF surface and its low Na${}^{+}$ ions intercalation performance.

## 2. Materials and Methods

Commercially available non-woven type graphitic carbon fibers (CF) made from poly-acrylonitrile (PAN) were used as a substrate [25]. The CF substrate was cut into 7 mm diameter pieces of ∼3 mm thickness. The single carbon fiber had diameter ranging from ∼10 to 25 μm. In the next step, carbon nanotubes were grown on CF substrate by a chemical vapor deposition (CVD) method using a methanol precursor (carbon source), nitrogen gas as a carrier gas, a Co:Mn (1:1 wt. ratio) catalyst, and methanol solvent. The CNTs were grown at 650 C temperature for 10 min in a tubular furnace. The weight of CNTs deposited from CVD process as measured for weight percentage (wt.%) calculation. Later on, electrochemical deposition for vanadium oxide (V${}_{2}$O${}_{5}$.nH${}_{2}$O) nanostructures on CNTs coated CF substrate, VOSO${}_{4}$.xH${}_{2}$O powder (0.6 g) was dissolved into 60 mL 4:1 v:v ratio of DI-water (80%) and ethanol (20%) (pH = 4) to prepare the electrolyte solution. After the electrochemical deposition V${}_{2}$O${}_{5}$.nH${}_{2}$O-CNTs-CF sample was washed in deionized water and dried for over night at 80 C in vacuum oven. To follow the electro-insertion of Mn${}^{2+}$ cations into V${}_{2}$O${}_{5}$.nH${}_{2}$O structure, the V${}_{2}$O${}_{5}$.nH${}_{2}$O-CNTs-CF were subjected to anodic polarization (+0.15 mA for one hour) in electrolyte prepared with 0.25 mol/liter of Mn(CH${}_{3}$COOH)${}_{2}$ in DI-water (pH = 6) to achieve the final product of MVO on CNTs coated CF substrates [26]. MVO-CNTs-CF electrodes were ready for further electrochemical characterization. The mass ratio or weight percentage (wt.%) of CNTs and active MVO material were calculated for comparative study.

**Figure 1:**
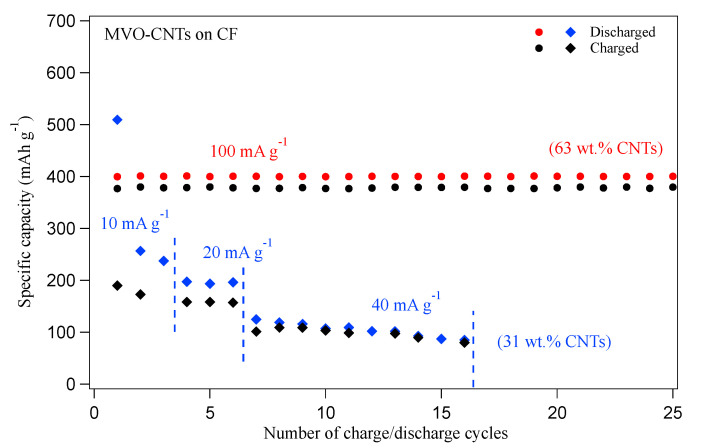
The specific capacity versus galvanostatic charge/discharge cycles at different current density values for the MVO-CNTs with 63 wt.% and 31 wt.% of CNTs electrodes, respectively. The MVO-CNTs electrode with 63 wt.% of CNTs sample data are taken from the Ref. [27].

The electrochemical characterization was performed in Swagelok-type cells using a polypropylene membrane (Celgard 2300) as a separator and metallic sodium as a counter and reference electrodes. Ethyl carbonate and propylene carbonate (EC:PC, 9:1, *v*/*v* 96 chameleon Reagent) were mixed with 1.0 mol L${}^{-1}$ NaPF${}_{6}$ for use as the electrolyte. The MVO-CNTs/CF electrode investigated in the present work was submitted to 20 discharge/charge cycles between 1.0 and 3.5 V vs. Na/Na${}^{+}$ at gravimetric density shown in Figure 1. However, as in the reference [26], two voltammetric cycles were applied in the same potential window scan-rate of 0.5 mV.s (MVO-CNTs as pseudo-capacitive behavior). The electrodes were thermally treated at 100 C temperature under reduced pressure for 12 h before the cells were assembled in an argon filled glove box.

Later on for post-mortem analysis, the MVO-CNTs pristine and Na${}^{+}$ cycled electrode samples were characterized via Scanning Electron Microscopy (ZEISS Gemini SIGMA 300), micro-Raman Spectroscopy using the HORIBA model, Ar-ion 532 nm laser light, 600 lines/mm grating system and Scanning Photoelectron Microscopy (SPEM) at ESCA Microscopy beamline 2.2 L, Elettra Synchrotron Trieste, respectively. Chemical mapping and core-level XPS spectra were acquired using a constant X-ray photon energy of 650 eV. The photon energy calibration was performed by taking Au4f core-level reference spectra on pure gold foil sample. Before the ESCA microscopy experiments, both electrodes were subjected to a cleaning process at mild temperature (∼100 C) in a UHV chamber.

Earlier, we found that the wt.% of CNTs into MVO-CNTs composite played an important role in the discharged capacity of Na${}^{+}$ ions intercalation/deintercalation process as shown in Figure 1. The MVO-CNTs incorporated 63 wt.% of CNTs endowed ∼400 mA h g${}^{-1}$ of discharge specific capacity between 1.0 and 3.5 V potential window and 100 mA g${}^{-1}$ current density as reported in the reference article [27]. While the CNTs in CNTs-carbon fiber (CF) electrode contributed only 10 mA h g${}^{-1}$ of discharge specific capacity within 3.5–0.2 V vs. Na/Na${}^{+}$ potential window at 0.1 A g${}^{-1}$ current density. Furthermore the CNTs mass loading was mitigated to ∼31 wt.% (meaning that electrodeposition of MVO nano-composite was in higher amount than in the case of 63 wt.%, i.e., the CNT mass is always the same in all electrodes) then the specific discharge capacity declined to approximately 100 mA h g${}^{-1}$ at 40 mA g${}^{-1}$ current density within 1.0–3.5 V potential window. In the MVO synthesis process the electrodeposited amount can vary thus the overall wt.% ratio of CNTs and MVO also varies from 30 to 70%. We considered CNTs wt.% comparable to MVO nano-composite mass for this study. The CVD deposited CNTs on CF surface consisted more or less similar mass after each deposition. The specific surface area of the electrodes was measured in a Quantachrome NOVA BET Surface Area Analyzer, using Brunauer-Emmett-Teller (BET) method for N${}_{2}$ adsorption isotherm, where we observed that MVO-CNTs sample had a high BET specific surface area compared to MVO-CF sample without CNTs coating on CF.

From our previous results, an optimized wt.% using the charge and mass associated with electrodeposition method incorporation with CNTs previously grown by CVD. The CNTs wt.% ratio into resulting MVO-CNTs was found as optimal in our previous work between 60 and 70%, provided a remarkable specific capacity. Under this condition we show in the SEM analysis of our previous work that the tubes morphology was visible. In the present work we investigate the effect of the CNTs loading percentage on the performance of the MVO-CNTs composite in terms of electronic structure and phase distribution on Na${}^{+}$ cycled sample.

## 3. Results

### 3.1. Morphological and Structural Phase Analysis

**Figure 2:**
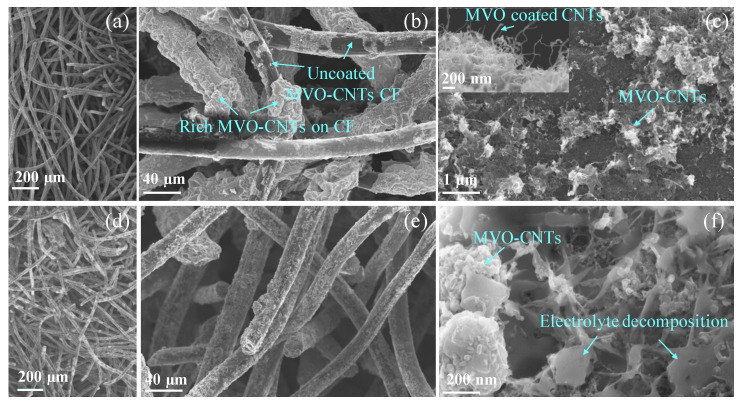
SEM images of pristine (**a**–**c**) and Na${}^{+}$ cycled (**d**–**f**) MVO-CNTs electrodes, respectively, on the CF non-woven type network. The inset in (**c**) shows the high-resolution SEM image of MVO coated CNTs network.

To understand the role of CNTs in MVO-CNTs composite after charge/discharge cycles, we have focused our attention on the electrode that present fading capacity (CNTs 31 wt.%).This one will reflect a higher concentration of oxides on the CNTs network, which can cover completely the CNTs network. Scanning electron microscope (SEM) images of pristine and Na${}^{+}$ cycled MVO-CNTs electrode with a weight fraction of 31 wt.% of CNTs are shown in Figure 2. The CF felt is a non-woven network (Figure 2a,d) with a thickness of around 3 mm which consists of entangled carbon fibers which were coated by CNTs using CVD technique. This kind of CF sizing can be applied without difficulty depending on the CVD reactor dimension including CF with higher thickness. The diameter of pristine and cycled MVO-CNTs/CF was measured in the range from ∼20 to 30 μm (Figure 2b,e). The SEM image of pristine MVO-CNT/CF (Figure 2b) shows the presence of a rich MVO deposition on the surface of CF only in the regions where the CNT network is a dense deposit. In fact, it is possible to find it in the CF uncoated by MVO (darker regions) in contrast with the regions of CF surface containing a high density of CNTs (Figure 2b). This occurs because the electrolyte wets more the CNTs than the upsizing fiber [28]. Furthermore, the high-resolution SEM image of the pristine electrode (Figure 2c, inset image) shows regions where it is possible to see an individual CNT whose surfaces are coated by MVO. The higher concentration of MVO into MVO-CNTs leads to formation of agglomeration of particles as well. Under this condition, the SEM analysis disclosed a clear non-uniform electrolyte decomposition as expected on the surface of electrode [29] (as shown by arrows) on the Na${}^{+}$ cycled MVO-CNTs (Figure 2f). After the Na${}^{+}$ ions intercalation cycles into MVO-CNTs nano-composite, it was observed the presence of flakes and other CEI deposits on the surface of the MVO-CNT network (Figure 2f). This suggests that CEI formation does not occur only on the surface of individual MVO-CNTs however it would occur on the MVO-CNTs with lower MVO concentration. In the case of 63 wt.% CNTs into MVO-CNTs it occurs where we cannot cover the entire CNTs network with MVO to obtain a good results. Figure 2f shows that the CEI deposits do interconnect the MVO-CNTs. This result suggests that the MVO decoration on CNTs/CF needs to avoid the formation of large deposits of MVO onto the surface of the CNTs network, otherwise the formed CEI on this kind of electrode will not be enough to avoid fading capacity and poor Na-specific capacity.

**Figure 3:**
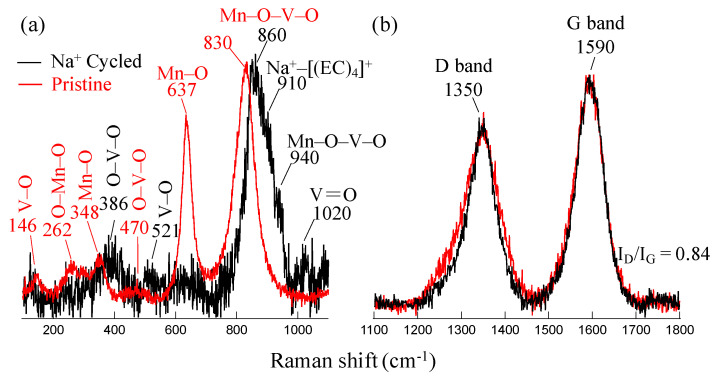
Micro-Raman scattering spectra of pristine and Na${}^{+}$ cycled MVO-CNTs electrodes in MVO region (**a**) and carbon bands region (**b**). The Raman spectra were normalized with respect to the highest peak intensity.

The electronic structure of the pristine and Na${}^{+}$ cycled MVO-CNTs electrodes were investigated by micro-Raman scattering spectroscopy as shown by Raman spectra in Figure 3a,b. The probing depth of incident laser light source (λ = 532 nm) was approximately in the range of 0.5–1.0 μm. Figure 3a shows the Raman spectra in the range of MVO Raman shift, where the peak centered in pristine MVO-CNTs sample spectra at ∼830 cm${}^{-1}$ (full width at half maximum (fwhm) = ∼60 cm${}^{-1}$) is assigned to O-Mn-O-V-O bonds stretching vibrational modes which is associated to β-Mn${}_{2}$V${}_{2}$O${}_{7}$ phase in monoclinic (c2m) symmetry [24,26]. Furthermore the lower Raman shift values at 146 and 470 cm${}^{-1}$ are assigned to [VO${}_{5}$-VO${}_{5}$] atomic layers sliding motion in x-y axis plane and O-V-O bending vibrational modes, respectively, in layered α-V${}_{2}$O${}_{5}$ phase [30]. Finally the Raman shifts values at 262, 348 and 637 cm${}^{-1}$ are associated to waving, bending (E${}_{g}$) and stretching (A${}_{1g}$) vibrational modes from O-Mn-O and Mn-O bonds mainly assigned to Mn${}_{2}$O${}_{3}$ phase [31,32]. Raman scattering revealed the presence of Mn${}_{2}$O${}_{3}$ rich β- Mn${}_{2}$V${}_{2}$O${}_{7}$ phase in pristine MVO-CNTs electrode sample. The investigations on the various samples it has been found that the MVO structural phase of pristine MVO-CNTs composite was independent from CNTs wt.% and confirmed always as Mn${}_{2}$O${}_{3}$ rich β- Mn${}_{2}$V${}_{2}$O${}_{7}$ phase [26].

Na${}^{+}$ cycled electrode sample shows a blue shift of 30 cm${}^{-1}$ in MVO phase peak which is associated with crystal structural distortion after the Na intercalation process and interaction of MVO-CNTs with electrolyte. The low intensity of Raman band suggests degradation of MVO which explains the Na-specific capacity with cycle number (Figure 1).

In the Na${}^{+}$ cycled MVO-CNTs electrode Raman spectra, the additional peaks at 386, 521, 1020 cm${}^{-1}$ and 940 cm${}^{-1}$ are associated to bridging bonds V-O-V, V-O (along the x–y axis plane) and V=O (along the c-axis) bond vibrations which are due to δ- V${}_{2}$O${}_{5}$ and Mn${}_{x}$V${}_{2\pm y}$O${}_{5\pm z}$ secondary phases formation, respectively [30,33]. Moreover, the Raman shift at 910 cm${}^{-1}$ is assigned to breathing vibrations (A${}_{1}$ mode) of Na-ions and ethyl carbonate (EC) molecules, i.e., [(Na(EC)${}_{4}$)]${}^{+}$ when the NaPF${}_{6}$ electrolyte dissolved into EC/EMC organic solution [34,35]. The low intensity of this Raman band is due to the weak interaction between Na${}^{+}$ ions and EC molecules. In the Na${}^{+}$ cycled electrode’s Raman spectra, the absence of Mn${}_{2}$O${}_{3}$ phase peak indicates the manganese oxide phase dissolution into electrolyte solution during charge/discharge polarization of the electrode. As a result, sodiation and de-sodiation could affect mainly the oxygen atoms occupancy in the MVO structure through the change of vanadium valence state from V${}^{4+/5+}$ to V${}^{3+}$ and manganese from Mn${}^{3+}$ to Mn${}^{2+}$ after an entire cycle. After Na${}^{+}$ cycle, the increase in A${}_{1g}$ frequency is related to the lower valence states of V and Mn in a more packed MVO structure.

Figure 3b shows the carbon bands, where the peaks at ∼1350 and 1590 cm${}^{-1}$ Raman shifts are assigned to D- band and G- band, respectively. D- band is associated to defective C-C bonds vibrational mode (A${}_{1g}$) mainly due to C-C bonds in sp${}^{3}$ hybridization, chemical decoration on C-C ring of oxide/hydroxide groups, or the presence of amorphous carbon. While the G- band shows sp${}^{2}$ hybridized C-C bonds vibrational mode (E${}_{2g}$) along circumferential and growth axis direction along CNTs tubes. The full-width half maximum (fwhm) values for D- and G- bands of pristine, Na${}^{+}$ cycled samples were calculated as 110, 79 and 84, 76 cm${}^{-1}$, respectively. Although the fwhm values of the carbon bands in Na${}^{+}$ cycled sample’s spectra were slightly lower than the pristine sample, the I${}_{D}$/I${}_{G}$ ratio was 0.84 for both electrode samples. These alterations in the MVO-CNTs Raman bands with discharge/charge cycles suggest a possible small alteration in the crystallinity associated with the stacking of graphene layers present in the CNTs walls. The CNTs used in the present study have walls that contain cup-stacked structures [26]. We believe that in the potential range where the MVO-CNTs was polarized, Na intercalation is very difficult to occur. Then we speculate that the alterations in the FWHM of D and G bands might be due to mechanical stress resulting from Na intercalation.

### 3.2. SPEM Maps and Electronic States Analysis

**Figure 4:**
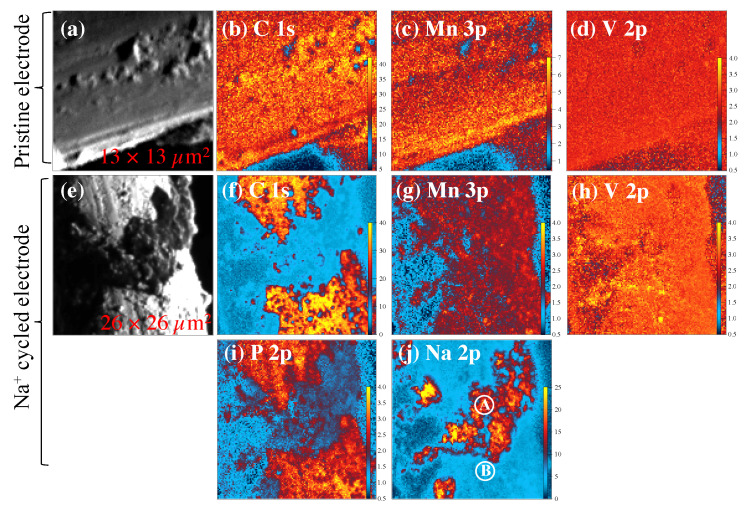
Scanning X-ray photoelectron microscopy (SPEM) maps of pristine (**a**–**d**) and Na${}^{+}$ cycled (**e**–**j**) MVO-CNTs electrodes, respectively, for selected elements. The color scale bar in each chemical SPEM map is representing the relative chemical concentration for the selected elements. The maps in gray color (**a**,**e**) are the raw SPEM maps before eliminating topographic features.

Scanning X-ray photoelectron microscopy (SPEM) maps are shown in Figure 4. SPEM is an advanced surface characterization technique where an incident X-ray beam has a probing depth of ∼1.0–2.0 nm with 130 nm spatial resolution.

Raw SPEM maps at C 1s core level energy in gray color as shown in Figure 4a,e show the topographic features of the MVO-CNTs on a CF surface. The colored SPEM maps are the chemical maps at the selected core level binding energies after eliminating the topographic features using background removal tool [36]. A uniform distribution with a relatively different chemical concentration of carbon, manganese and vanadium elements were observed as shown in Figure 4a–d.

From the carbon and manganese SPEM maps (Figure 4b,c), it is possible to see that carbon-rich regions show low manganese concentration and vice versa, while the vanadium is quite uniformly distributed. This means that a sizable amount of manganese oxide phase was electrochemically grown on hydrated V${}_{2}$O${}_{5}$.nH${}_{2}$O previously electrodeposited on the CNTs surface. The manganese shows higher chemical concentration than vanadium (see color scale bar) on the pristine MVO-CNTs sample as found in Raman scattering results (Figure 3a). Furthermore in the SPEM maps of Na${}^{+}$ cycled sample Mn 3p and V 2p show a uniform chemical distribution. The lower presence of manganese (Figure 4g) than that of vanadium (Figure 4h) element is evidence of manganese oxide phase’s dissolution in Na${}^{+}$ cycled sample. The strong signal of vanadium visible in V 2p map is consistent with the presence of secondary vanadium oxide phases which are compatible with Raman scattering results. The decomposition of phosphorus phases was favored on the carbon fiber’s surface where a discontinuous MVO-CNTs coating was present as shown in Figure 4f,i. On the other hand sodiated chemical phases were found on MVO-CNTs coating region (Figure 4j). This means that the hydrophobic amorphous carbon fibers did not host the Na${}^{+}$ ions into their structure and formed the carbon–phosphorus-based chemical phases on the surface. Metal phosphides were also formed as identified by core-level XPS spectra on both regions (i.e., rich and poor carbon, phosphorus and sodium regions) explained in the text below.

**Figure 5:**
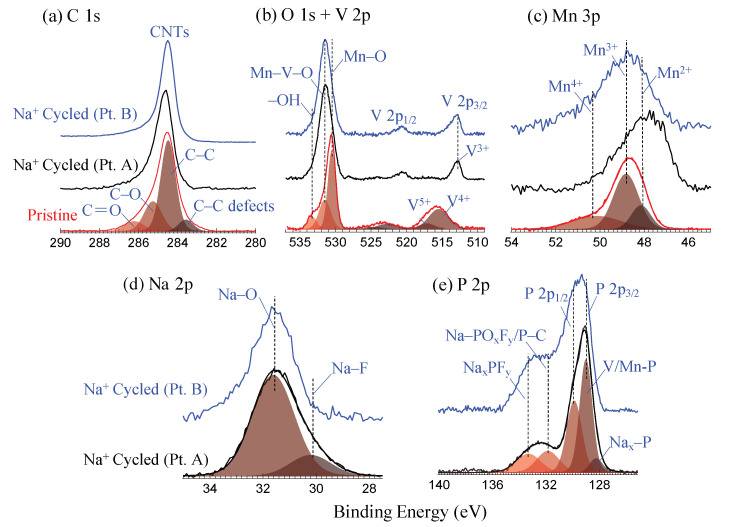
X-ray photoelectron spectroscopy (XPS) core level spectra of C 1s (**a**), O 1s + V 2p (**b**), Mn 3p (**c**), Na 2p (**d**) and P 2p (**e**). Point A (Pt. A) and Point B spectra were acquired on the positions as shown on SPEM map in Figure 4j on Na${}^{+}$ cycled sample. XPS core-level spectra intensity are shown as normalized.

Figure 5 shows the high-resolution XPS spectra for the detailed electronic structure investigation. The reported XPS spectra were acquired on the uniform region for pristine sample (Figure 4a–c), and defined locations at points A, B in the case of cycled electrode sample (Figure 4j) as shown in SPEM maps. The deconvoluted components of C 1s core level spectra (Figure 5a) at binding energies (BEs) 283.5, 284.5, 285.2 and 286.2 ± 0.1 eV are assigned to C-C defects in CNTs, sp${}^{2}$ C-C bonds, C-O/O-C-O and C=O bonds, respectively [37,38,39,40,41,42]. C 1s core level spectra at point A location (on cycled sample) shows the binding energy shift of +0.2 eV which can be due to Na${}^{+}$ ions trapped into multi-walls of CNTs. In the V 2p${}_{3/2}$ core level spectra (Figure 5b), the pristine sample showed the mixed valance states V${}^{4+/5+}$ at the binding energies of 515.5 and 517.1 eV, while the electrochemically reduced V${}^{3+}$ state (BE = 512.9 eV, Δ = 7.7 eV) was observed in cycled MVO-CNTs sample on both points A and B [43]. Furthermore in O 1s core level spectra the BEs at 530.6 and 533.2 eV are assigned to Mn-O (coordinated with V atoms) and -OH chemical bonds respectively [44,45], which is consistent with previous reported Raman scattering results and C 1s core level spectra of pristine sample. The component of MVO phase appeared at BE of 531.4 eV in O 1s core level spectra. From the Mn 3p core level spectra (Figure 5c) the mixed Mn${}^{2+/3+/4+}$ valence states were present in both pristine and cycled samples. The Mn${}^{3+}$ oxidation state (BE = 48.7 ± 0.1 eV) is related to Mn${}_{2}$O${}_{3}$ phase, while Mn${}^{2+}$ (BE = 47.7 eV) comes from the MnOOH${}^{-}$ on the MVO-CNTs surface and Mn${}_{2}$V${}_{2}$O${}_{7}$ (MVO) phase. On the cycled sample the chemical phases of Na${}_{2}$O (Na-O) and NaF${}_{2}$ (Na-F) phases [11,34] at BE of 31.6 and 30.1 eV, respectively, were observed as shown by Na 2p core level spectra in Figure 5d. Finally in P 2p${}_{3/2}$ core level spectra (Figure 5e), Na${}_{x}$-P, metal phosphides (V-P, Mn-P), Na-PO${}_{x}$F${}_{y}$/P-C and Na${}_{x}$PF${}_{y}$ chemical bonds were identified at BE of 128.2, 129.0, 129.3, 132.5 and 133.6 eV, respectively, [11,46,47]. The multiplet splitting (P 2p${}_{3/2}$− P 2p${}_{1/2}$) for P 2p core-level spectra was as 0.82 eV.

## 4. Discussion

From an analysis of the Raman scattering and SPEM results of the Na${}^{+}$ cycled electrode, the formation of manganese–fluoride (MnF${}_{2}$), metal–phosphates, and dissolution of transition metal oxide (Mn${}_{2}$O${}_{3}$) were the main factors identified behind the loss of discharge capacity in MVO-CNTs binder-free carbon-fiber-based sodium ion battery. The dissolution of manganese oxide may have occurred due to hydrogen fluoride (HF) formation which reacts with the sodium metal anode surface and CEI layer resulting in the formation of a discontinuous CEI layer. The CEI morphology is expected to have an influence on the Mn oxide dissolution from MVO. In the case of MVO-CNTs with CNTs 63 wt.%, the eventual dissolution of MVO cannot be disregarded although to a smaller degree. The electrochemical reduction of vanadium from V${}^{4+}$ to V${}^{3+}$ electronic states and blue shift of MVO Raman peak are evidence of the reversible intercalation process of Na${}^{+}$ ions.The MVO phase modification with structural distortion occurred after certain Na${}^{+}$ charge–discharge cycles as observed by Raman spectra. Nevertheless, the resulting MVO phase leads to MVO-CNTs which provide lowest discharge/charge Na specific capacity (Figure 1). A similar phenomenon was observed after Zn${}^{+}$ ions intercalation/de-intercalation into MVO phase in Zn-ion battery where the crystal structure of MVO was found quite stable after Mn dissolution [33,48]. Additionally, the presence of secondary phases of Mn${}_{x}$V${}_{2}$O${}_{5}$ and V${}_{2}$O${}_{5}$ in Na${}^{+}$ cycled electrode were also able to host Na${}^{+}$ ions reversibly into their layered structure and may contribute in discharge capacity. The PO${}_{x}$F${}_{y}$ and phosphorus-carbonate (P-C) phases in the CEI layer were increased where the CNTs coating was low and these components favour stable discharge capacity. The presence of sodium and manganese fluoride (NaF/NaF${}_{2}$, MnF${}_{2}$) chemical phases are one of the reasons behind the manganese oxide loss resulting in lower discharge capacity and Na${}^{+}$ ions trapping. The uniform photoelectron signals detected from beneath the CEI layer are evidence of a thin (≤1 nm) layer formation which is found comparatively thinner than that on other binder-based transition metal oxide cathodes.

## 5. Conclusions

Our results show that CNTs played a crucial role in the formation of CEI on the MVO-CNT/CF network structure and its phase stability. Practically, it is not possible to observe the shape of CNTs on the surface of CF, following electrodeposition of MVO on its surface. The MVO-CNTs/CF electrode with a high concentration of MVO is expected to be more resistive, thus inferring that the Na-specific capacity will also suffer. This occurs because a sufficient amount of CNTs provides a more complex electronically conductive network for electrochemical deposition of MVO nanoparticles. In turn, this provides a rich and more uniform MVO-CNTs coating with reduced particle size on the CF surface. Another reason for the addition of optimized CNTs wt.% with MVO is that CNTs are capable of hosting the Na${}^{+}$ ions, besides its role in the CEI formation, such that the CNTs type cannot be ignored. The SPEM maps confirmed that the CEI layer thickness was ≤1 nm on the binder-free MVO-CNTs electrode surface from the conventional NaPF${}_{6}$ electrolyte. MVO-CNTs composite provides a stable and uniform electronic structure phase following the Na${}^{+}$ charge–discharge cycles. The optimization of active transition metal oxide, i.e., MVO and wt.% of additives such as carbon nanotubes, deserves more attention regarding enhancements in electrochemical performance and life-span of rechargeable Na${}^{+}$ ion batteries. The promising MVO-CNTs composite can be applied across various fields of wearable electronics devices, multi-cation batteries, sensors and hydrogen production for fuel cells. The MVO-CNTs/CF electrodes are binder-free; however, they can be converted to material binder electrodes through the usage of a mill. Therefore, this study might be useful to the development of another nano-composite type with or without the support of NTs on CF.

## Data Availability

Data are available upon reasonable request.

## Abbreviations

The following abbreviations are used in this manuscript:

CNTs	Carbon Nanotubes
CF	Carbon Fiber
CEI	Cathode Electrolyte interphase
MVO	Manganese Vanadium Oxide
NIBs	Sodium-ion Batteries
SEI	Solid Electrolyte interphase
SPEM	Scanning Photoelectron Microscopy
